# Involuntary Proving of Arnica

**Published:** 1885-11

**Authors:** E. W. Berridge

**Affiliations:** London


### INVOLUNTARY PROVING OF ARNICA.

E. W. BERRIDGE, M. D., LONDON.

The following report was sent me by a patient: He received a blow on left cheek from a cricket ball, which blackened the eye and whole side of face. Applied a strong dilution of *Arnica*, and took one or two drops internally at once, and continued to do so for two or three days. The swelling then became red, puffy, and angry, especially under the eye, with unmistakable symptoms of erysipelas, scaly and in spots, extending down the cheek and on the forehead. *Hamamelis* and *Calendula* had little or no effect. Then, as the swelling seemed exactly like the sting of a bee under the eye, he tried *Apis*, first decimal, in two-drop doses once or twice, with immediate and striking relief, the swelling going down within half an hour. Did not repeat the dose till next day, when the swelling seemed to return slightly, and the same result followed taking the medicine. A stye then formed on lower eyelid, and *at once* the right finger began to pain, ending in a gathering, which discharged. Was the whitlow the effect of *Apis?*

